# In-cell NMR: a topical review

**DOI:** 10.1107/S2052252516020625

**Published:** 2017-02-15

**Authors:** Enrico Luchinat, Lucia Banci

**Affiliations:** a Magnetic Resonance Center – CERM, University of Florence, Via Luigi Sacconi 6, 50019 Sesto Fiorentino, Florence, Italy; b Department of Biomedical, Clinical and Experimental Sciences, University of Florence, Viale Morgagni 50, 50134 Florence, Italy; c Department of Chemistry, University of Florence, Via della Lastruccia 3, 50019 Sesto Fiorentino, Florence, Italy

**Keywords:** in-cell NMR, nuclear magnetic resonance, cellular structural biology, cellular environment, protein interactions

## Abstract

Classical structural biology approaches allow structural characterization of biological macromolecules *in vitro*, far from their physiological context. Nowadays, thanks to the wealth of structural data available and to technological and methodological advances, the interest of the research community is gradually shifting from pure structural determination towards the study of functional aspects of biomolecules. Therefore, a cellular structural approach is ideally needed to characterize biological molecules, such as proteins, in their native cellular environment and the functional processes that they are involved in. In-cell NMR is a new application of high-resolution nuclear magnetic resonance spectroscopy that allows structural and dynamical features of proteins and other macromolecules to be analyzed directly in living cells. Owing to its challenging nature, this methodology has shown slow, but steady, development over the past 15 years. To date, several in-cell NMR approaches have been successfully applied to both bacterial and eukaryotic cells, including several human cell lines, and important structural and functional aspects have been elucidated. In this topical review, the major advances of in-cell NMR are summarized, with a special focus on recent developments in eukaryotic and mammalian cells.

## Abstract 2

In-cell NMR has emerged in the past decade as a unique approach to obtain structural and functional information on biological macromolecules within living cells at atomic resolution. In this review, the major advances of in-cell NMR are discussed, with a special focus on recent developments and applications in eukaryotic and mammalian cells.

## 1. Introduction   

The structure of biological macromolecules is critical to understanding their function, their mode of interaction and relation with their partners, and how physiological processes are altered by mutations or changes in the molecular environment. Detailed structural information is especially needed for drug and vaccine design. Since the decoding of the genomes of several organisms, with the most relevant being the human genome, large efforts have been undertaken to solve novel protein structures, often within specific structural genomic projects with a defined focus.

Since the advent of structural biology, X-ray crystallography and nuclear magnetic resonance (NMR) have been the only two techniques that are able to provide structural information at atomic resolution. Today, most macromolecular structures are still obtained by X-ray crystallography, which is the most robust method, provided that well diffracting crystals can be obtained, while NMR has proven to be an invaluable tool to investigate macromolecular structure and dynamics in aqueous solution at room temperature. Solution NMR is especially powerful for the investigation of protein–ligand and protein–protein interactions, in particular those of a transient nature, binding constants, folding thermodynamics and kinetics. In addition, solid-state NMR has seen increasing application in determining the structures of insoluble biological aggregates that are difficult to crystallize, such as fibrils, viral capsids and membrane proteins.

In the past decade, technical developments in cryo-electron microscopy (cryo-EM) have also made it possible to obtain structures of large macromolecular complexes at quasi-atomic resolution. Indeed, the recent progress of cryo-EM in terms of decreasing the molecular-size limit and increasing resolution have now enabled the structural characterization of previously inaccessible, hard-to-crystallize large systems. Currently, advanced techniques are being developed that could in the future allow protein structures to be calculated from single-molecule diffraction data by exploiting the extremely high brilliance of X-ray free-electron lasers (XFELs).

In addition to purely structural methods, hybrid method­ologies have been developed in which one or more high-resolution techniques are combined with other biophysical approaches such as small-angle X-ray/neutron scattering (SAXS/SANS), chemical cross-linking and mass spectrometry to uncover the structure and dynamic properties of complex multi-component systems.

Nowadays, partly thanks to the wealth of structural data that are already available, interest has focused more on the functional characterization of biological macromolecules. As a consequence, classical structural biology research needs to interface more with cellular biology, as it is crucial for the structural data obtained *in vitro* to be validated within the cellular or tissue context. Hence, a true ‘cellular structural biology’ approach should allow macromolecules to be characterized directly in their native environment. Ideally, such an approach would guarantee the high significance of data obtained *in vivo* or in the cell with the high resolution of a structural technique.

In the last decade, NMR spectroscopy has been applied to obtain structural and functional information on biological macromolecules inside intact, living cells. The approach, termed ‘in-cell NMR’, leverages the improved resolution and sensitivity of modern high-field NMR spectrometers and exploits increased levels of the molecule(s) of interest selectively enriched with NMR-active isotopes (*e.g.* ^13^C and ^15^N). This approach differs from the previously developed ‘*in vivo* NMR’ applications, where lower resolution, homonuclear NMR had been applied to living cells and organisms to study naturally abundant small molecules and metabolites.

Since its inception, in-cell NMR has gradually emerged as a possible *trait d’union* between structural and cellular approaches. Being especially suited to investigate the structure and dynamics of macromolecules at atomic resolution, in-cell NMR can fill a critical gap between *in vitro*-oriented structural techniques, such as NMR spectroscopy, X-ray crystallography and single-particle cryo-EM, and ultrahigh-resolution cellular imaging techniques, such as super-resolution microscopy and cryo-electron tomography, which have seen impressive development in recent years.

In this topical review, we summarize the major advances of in-cell NMR since its first application, and we further report the recent developments of this promising methodology, with a special focus on its application to study proteins in eukary­otic and mammalian cells and on the development of cellular solid-state NMR approaches.

## 2. Overview of in-cell NMR approaches   

The first examples of in-cell NMR were reported in *Escherichia coli* cells. Serber and coworkers showed that small globular proteins could be overexpressed in *E. coli* and isotopically labelled to a sufficient level that it was possible to detect them above the other cellular components by heteronuclear NMR (Serber *et al.*, 2001 ▸). This approach was demonstrated for the N-terminal metal-binding domain of bacterial mercuric ion reductase (NmerA) and on human calmodulin. Since then, bacteria have proven to be a suitable organism for in-cell protein NMR studies. The generally adopted protocol consists of a two-step culture, in which bacteria are first grown in unlabelled medium and then transferred into fresh, isotopically labelled minimal medium, where protein expression is induced. Such an approach exploits the existing biotechnological tools for recombinant protein expression in *E. coli*, such as efficient expression vectors for high protein yield, independent induction systems for controlled sequential expression of two or more proteins (Burz *et al.*, 2006 ▸; Burz & Shekhtman, 2008 ▸) and the possibility of using auxotrophic strains to perform amino-acid-selective labelling (Serber *et al.*, 2004 ▸; Banci *et al.*, 2011 ▸). Typically, uniform ^15^N labelling is preferred to uniform ^13^C labelling owing to lower sample-preparation costs and better selectivity for the NMR signals with respect to the cellular background. However, amino-acid-selective labelling strategies can show advantages with ^13^C, as was shown by (methyl-^13^C)methionine labelling, which provided good selectivity against the cellular background (Serber *et al.*, 2004 ▸). In addition to ^15^N and ^13^C, ^19^F has also been utilized to probe protein dynamics in bacteria (Li *et al.*, 2010 ▸; Ye *et al.*, 2013 ▸). ^19^F is a 100% abundant and high-sensitivity isotope, which can be incorporated into the protein of interest by the use of non-natural ^19^F-amino acids. As biological molecules do not contain F atoms, the resulting ^19^F NMR spectra are virtually background-free.

While in-cell NMR in bacterial cells is becoming a relatively straightforward methodology, bacteria may not be an appropriate model system for studying eukaryotic proteins. Ideally, these proteins should be characterized in a cellular environment which matches the native one as closely as possible. The bacterial cytoplasm may lack the machinery to correctly fold a eukaryotic protein and allow its maturation. Moreover, any functional partner will be absent, and the protein will not be correctly targeted to its physiological localization. In the first efforts to establish eukaryotic model systems for in-cell NMR, *Xenopus laevis* oocytes were employed (Sakai *et al.*, 2006 ▸; Selenko *et al.*, 2006 ▸). In this approach, the isotopically labelled protein of interest is recombinantly expressed and purified from bacteria, and is delivered to the oocytes by microinjection. This method allows excellent labelling selectivity, as the labelled protein is introduced in unlabelled cells, effectively eliminating background NMR signals. However, it requires the protein solution to be highly concentrated in order not to dilute the content of the oocyte cytoplasm. Microinjection in *X. laevis* oocytes has also been applied to observe nucleic acids, which unlike proteins cannot be produced *in situ* at sufficient concentrations (Hänsel *et al.*, 2009 ▸). Using this approach, the conformation of G-quadruplex DNA structures was investigated in the physiological environment and was found to diverge from the topologies observed *in vitro* (Hänsel *et al.*, 2011 ▸, 2013 ▸).

A breakthrough in the methodological development of in-cell NMR came in 2009, when two research groups reported the observation of a labelled protein in human cells by NMR (Inomata *et al.*, 2009 ▸; Ogino *et al.*, 2009 ▸). Inomata and coworkers exploited a cell-penetrating peptide (CPP) of viral origin (from the HIV-1 Tat protein), which they fused to a mutated variant of human ubiquitin. The chimeric protein was able to translocate through the plasma membrane of cultured HeLa cells and to accumulate in the cytoplasm. Alternatively, the CPP peptide could be covalently attached to the protein surface through a disulfide bond, which could be cleaved in the reducing environment of the cytoplasm, releasing the free protein (Inomata *et al.*, 2009 ▸). Ogino and coworkers adopted a different strategy to deliver the protein of interest to human cells. By exploiting streptolysin O, a streptococcal pore-forming toxin, the authors could reversibly permeabilize the cell membrane by inducing the formation of pores through which the protein could translocate. The plasma membrane could then be re-sealed by treatment with Ca^2+^ to prevent cell death (Ogino *et al.*, 2009 ▸).

More recently, an additional method to deliver an exogenous protein for NMR in mammalian cells was developed which relies on electroporation. Cell electroporation was initially developed to efficiently transfect cells with nucleic acids by applying strong pulsing electric fields to permeabilize the plasma membrane. Selenko and coworkers showed that proteins could also be delivered to the cells by electroporation at a sufficient concentration to enable in-cell NMR (Binolfi *et al.*, 2016 ▸; Theillet *et al.*, 2016 ▸). This approach allows greater variability in terms of the required properties of the protein of interest and the type of cell line used, and will likely contribute to extend the applicability of in-cell NMR to more biological systems and cell lines.

**Figure 1:**
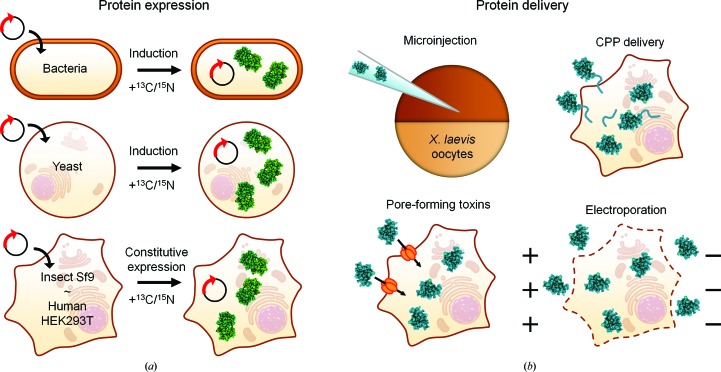
Schematic overview of the different in-cell NMR approaches. (*a*) Proteins (green) can be endogenously expressed and isotopically labelled in bacteria, yeast, insect and mammalian cells by introducing a suitable expression vector containing the gene of interest. Isotopically enriched media are provided after inducing protein expression/after transfection. (*b*) Exogenous proteins (blue) can be delivered to *X. laevis* oocytes by microinjection or to human cells exploiting either cell-penetrating peptides (CPP), cell permeabilization by pore-forming toxins or electroporation.

In addition to the protein-delivery approaches, which require heterologous protein production and extensive sample manipulation before insertion, intracellular expression has also been shown to be a viable approach for protein in-cell NMR in eukaryotic cells. Protein-expression strategies have been successfully applied to yeast (Bertrand *et al.*, 2012 ▸) and insect cells (Hamatsu *et al.*, 2013 ▸). Finally, our research group has shown that existing technologies for mammalian protein expression (Aricescu *et al.*, 2006 ▸; Seiradake *et al.*, 2015 ▸) can be adapted to produce samples of human cells suitable for in-cell NMR (Banci, Barbieri, Bertini *et al.*, 2013 ▸; Barbieri *et al.*, 2016 ▸). Compared with protein delivery, this approach has the advantage of allowing proteins to be studied directly in the cells where they are synthesized, without requiring any purification or chemical treatment prior to import. This strategy is especially beneficial for proteins that are prone to aggregation and/or are sensitive to the redox properties of the environment, and is ideally applied to the study of protein folding, maturation and other processes that occur after protein synthesis. Additionally, *in organello* NMR approaches are possible, in which protein expression is targeted to different cellular compartments by fusing specific targeting sequences to the protein of interest, such as a mitochondrial targeting sequence, as shown by our research group (Barbieri *et al.*, 2014 ▸). Moreover, two or more proteins can be expressed, with only one selectively labelled, by controlling the timing of expression (Luchinat *et al.*, 2016 ▸). The various approaches for in-cell NMR are summarized in Fig. 1 ▸.

## 3. Cellular solid-state NMR and DNP   

In parallel to solution NMR, solid-state magic angle spinning (MAS) NMR has been applied to investigate structural features of proteins in their native cellular environment. MAS NMR is not intrinsically limited by the slow rotational diffusion of the molecule and therefore is ideally applicable to the study of nonsoluble systems such as membrane proteins, large protein complexes or protein aggregates. Cellular applications of MAS NMR are challenging owing to its lower sensitivity compared with solution NMR, the need for selective labelling strategies to overcome the large cellular background, and cell sample-integrity issues. Solid-state NMR was first applied to study proteins in bacteria: Dötsch and coworkers showed that cellular proteins engaged in large complexes could be detected by solid-state NMR on frozen *E. coli* cells (Reckel *et al.*, 2012 ▸), while Baldus and coworkers applied solid-state NMR to obtain structural information on the abundant bacterial outer membrane protein OmpA inside intact *E. coli* cells and isolated native membranes (Renault, Tommassen-van Boxtel *et al.*, 2012 ▸). The sensitivity of MAS NMR can be greatly enhanced by exploiting the enhancement of dynamic nuclear polarization (DNP) through the use of paramagnetic agents. Using this approach, Baldus and coworkers obtained NMR signals from an overexpressed membrane protein (PagL) in intact cells and membrane fractions and could also detect other naturally abundant bacterial proteins and nucleotides (Renault, Pawsey *et al.*, 2012 ▸). More recently, Ramamoorthy and coworkers applied DNP to characterize the membrane-anchored cytochrome *b*_5_ in *E. coli* cells and in reconstituted bicelles (Yamamoto *et al.*, 2015 ▸). An exciting development in DNP enhancement is the selective hyperpolarization of the protein of interest by linking the paramagnetic moiety to a specific ligand, which allows the detection of proteins at very low levels with almost no background signal, as shown by Etzkorn and coworkers in bacterial cell lysates (Viennet *et al.*, 2016 ▸).

Unlike soluble proteins, the natural environment of membrane proteins can mostly be preserved by isolating native cellular membranes enriched with the protein of interest. This alternative approach, first demonstrated by Tian and coworkers (Fu *et al.*, 2011 ▸), provides higher resolution and sensitivity compared with intact cells, and ensures increased sample stability. Very recently, the Baldus group applied DNP solid-state NMR to characterize the soluble domain of the epidermal growth factor receptor (EGFR) in native membrane vesicles isolated from human cells (Kaplan *et al.*, 2016 ▸). The authors exploited the fact that the vesicles extracted from A431 cells are naturally enriched in endogenous EGFR, and produced ^13^C,^15^N-labelled samples by growing the cells on algae-derived media as a viable source of isotopically enriched nutrients (Fuccio *et al.*, 2016 ▸). The extracellular domain of EGFR was found to be highly dynamic in the unbound state, whereas EGF binding reduced the overall conformational entropy, likely promoting protein dimerization. The same group has recently developed labelling schemes based on fractional protonation in a deuterated background, which further improve the sensitivity and selectivity when studying proteins in native membranes and in principle can be applied to whole cells (Medeiros-Silva *et al.*, 2016 ▸).

While the previous applications were focused on membrane proteins, cellular solid-state NMR is also a powerful approach to study intracellular protein aggregates. Lindquist and coworkers characterized the folding state of the yeast protein Sup35 in cell lysates by DNP solid-state NMR (Frederick *et al.*, 2015 ▸). The purified labelled NM region of Sup35 adopted the amyloid state when incubated in unlabelled yeast lysates containing the prion form (PSI^+^), which acted as a template, thus allowing structural characterization in a close-to-native environment. Remarkably, a region of Sup35 that is intrinsically unfolded *in vitro* was shown to have higher propensity for β-sheet secondary structure in the cell lysates, likely as a consequence of interactions with intracellular partners. These recent applications prove that DNP-enhanced cellular solid-state NMR is a promising approach to characterize the structure and dynamics of challenging macromolecules under biologically relevant conditions.

## 4. Solution structure determination in living cells   

To date, in-cell NMR is the only technique that allows the determation of atomic resolution structures of proteins within an intact cellular environment. While this capability may not be revolutionary *per se* (indeed, a protein structure determined *in vitro* is conserved to a large extent in the cellular environment!), it will prove extremely useful in all instances where structural perturbations induced by interactions with the cellular environment modulate protein function.

In 2009, Sakakibara and coworkers solved *de novo* the structure of a bacterial metal-binding domain in *E. coli* cells (Sakakibara *et al.*, 2009 ▸). The authors exploited different labelling strategies, including ^13^C–^15^N labelling for backbone assignment and selective methyl-^13^C labelling for side-chain assignment, and collected spatial restraints using ^13^C- or ^15^N-filtered three-dimensional NOESY experiments. The short sample lifetime does not allow typical high-dimensionality NMR experiments to be recorded. In order to overcome this limit, the authors relied on nonlinear sparse-sampling schemes (Hoch *et al.*, 2014 ▸) to reduce the acquisition time of the three-dimensional experiments, and started from a fresh sample after each experiment.

**Figure 2:**
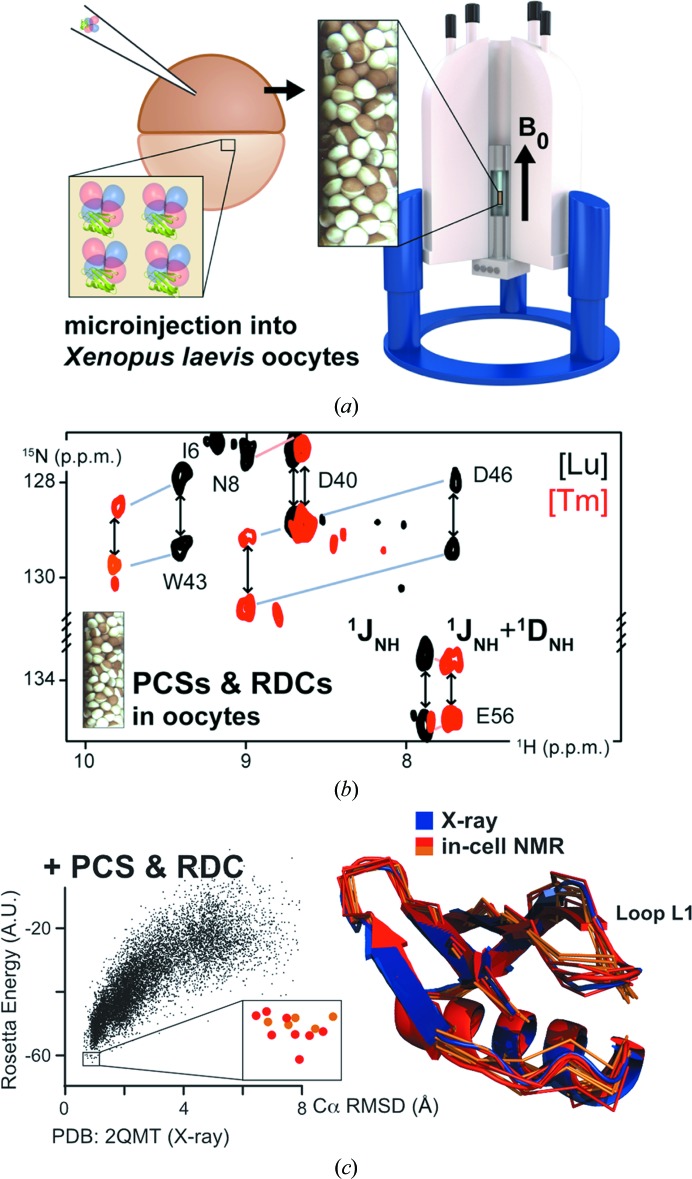
Structure calculation by paramagnetic in-cell NMR. (*a*) Preparation of a sample of oocytes injected with GB1 tagged with a paramagnetic lanthanide ion. (*b*) Overlay of in-cell NMR spectra showing signals from GB1 tagged with a diamagnetic (black) and a paramagnetic (red) lanthanide ion; structural restraints are calculated from the paramagnetic effects (PCSs and RDCs). (*c*) Scatter plot of *GPS-Rosetta* energy scores and C^α^ r.m.s.d. of GB1 models calculated with PCS and RDC input data (left) and lowest energy in-cell GB1 models compared with the X-ray structure of GB1 (right). Reprinted (adapted) with permission from Müntener *et al.* (2016 ▸). Copyright (2016) American Chemical Society.

The possibilities offered by in-cell NMR gained widespread recognition after the aforementioned structure was calculated exclusively from in-cell NMR data. In practical applications, however, the protein structure obtained *in vitro* is usually taken as a reference to interpret in-cell NMR data, as the information required for the structure calculation requires significant efforts in time and sample preparation. Very recently, an alternative approach has been independently proposed by two research groups (Müntener *et al.*, 2016 ▸; Pan *et al.*, 2016 ▸), which allowed the determination of intracellular protein structures in *X. laevis* oocytes (Fig. 2 ▸). In this approach, the protein of interest is chemically modified *in vitro* by attaching specifically designed tags that tightly bind a paramagnetic lanthanide ion (Otting, 2010 ▸; Keizers & Ubbink, 2011 ▸) and is subsequently delivered to the oocytes. Paramagnetic NMR effects, such as pseudo-contact shifts (PCSs) and paramagnetic residual dipolar couplings (pRDCs), can be measured with relatively little effort by comparing two-dimensional in-cell NMR spectra of the protein with the paramagnetic tag with reference spectra collected from the same protein with a diamagnetic tag. The paramagnetic effects measured for each nucleus can be converted to distance restraints from the lanthanide ion (PCSs) and angular restraints with respect to the paramagnetic (PCSs) or protein-alignment (pRDCs) tensors (Bertini *et al.*, 2002 ▸). Such restraints are used as input for *GPS-Rosetta* (Pilla *et al.*, 2016 ▸), which integrates them into a fragment-based *ab initio* structure calculation. This hybrid strategy does not require lengthy three-dimensional in-cell NMR experiments to be recorded, and only relies on the amide resonance assignment, which can be obtained *in vitro* and transferred to the in-cell NMR spectra. Both research groups demonstrated this approach using the same protein (the B1 domain of the staphylococcal protein G; GB1), and in both cases the calculated three-dimensional conformers were in good agreement with the solution structure of GB1 obtained *in vitro*. Notably, different paramagnetic tags were used, which were attached to GB1 in different positions, further demonstrating the robustness of this approach. This strategy is likely to prove extremely useful in the near future, especially when combined with the recent advancements in protein delivery. Indeed, in recent work a similar paramagnetic lanthanide tag was attached to ubiquitin, which was then delivered into the cytoplasm of HeLa cells by electroporation, allowing the authors to observe PCSs on the in-cell NMR spectra (Hikone *et al.*, 2016 ▸).

The use of paramagnetic NMR to obtain intracellular structural restraints benefits from the last-generation lanthan­ide binding tags, which are rigid and stable in the reducing environment of the cell, and from the fact that the range of accessible distances can be tuned by choosing different lanthanide ions. Therefore, this methodology will prove to be extremely useful in the near future, as it can in principle be applied to characterize protein–protein complexes in mammalian cells.

## 5. Biological insights   

In-cell NMR has the unique ability to provide atomic-scale data on the effect of the cellular environment on a protein. The intracellular environment is much more complex than most typical aqueous buffers used to characterize proteins *in vitro*. As an example, the bacterial cytoplasm contains around 300 g of proteins per litre, which make up to 25% of the total volume, and around 100 g of nucleic acids per litre, in addition to small solutes and ions. This complexity is reflected in the structural and dynamic properties of other macromolecules such as proteins. The main consequence of a high concentration of macromolecules in solution is molecular crowding, which acts through two main effects: excluded volume and intermolecular interactions. Both these features of the cytoplasm affect the thermodynamic properties of proteins by changing their folding landscape.

The excluded-volume effect increases the thermodynamic activity of a solute and, in the case of proteins, tends to favour more compact, folded states, as was shown to occur for the intrinsically disordered protein FlgM (from *Salmonella typhimurium*) in *E. coli* cells by Pielak and coworkers (Dedmon *et al.*, 2002 ▸). It is likely that the shift towards folded protein states is not complete and the disordered form is still present in the cytoplasm, as the same group showed recently (Smith *et al.*, 2015 ▸). Intermolecular interactions are harder to predict, as their consequences are highly variable among different proteins.

**Figure 3:**
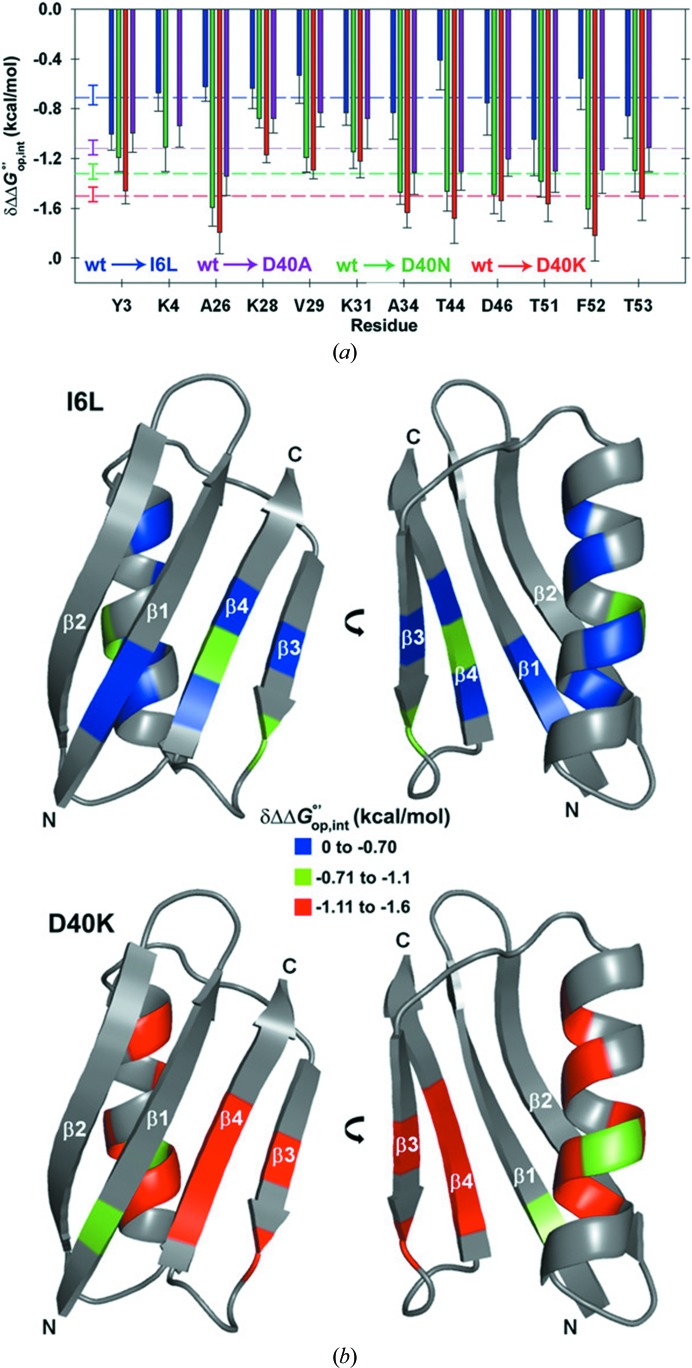
In-cell protein folding thermodynamics and quinary interactions. (*a*) Free energies of protein–cytosol interaction calculated for GB1 mutants with different net charges. (*b*) The quinary interactions calculated for each residue are larger for a charge-changing mutation (D40K, bottom) than for a neutral mutation (I6L, top). Adapted from Monteith *et al.* (2015 ▸).

The effects of intermolecular interactions are thought to add an additional layer of complexity to the classical concept of ‘structure determines function’, and their biological significance was previously postulated by E. H. McConkey, who coined the term ‘quinary structure’ (*i.e.* the next level of structure after quaternary). Interactions with other macromolecules were found to counteract the excluded-volume effect and, in some cases of natively unstable proteins, can shift the protein-folding equilibrium towards less compact states, as shown by Schlesinger *et al.* (2011 ▸). Since this striking example, the Pielak group has provided extensive data on the thermodynamics of protein folding as a function of intermolecular interactions within the bacterial cytoplasm. By measuring the hydrogen–deuterium (H–D) exchange rates of the amides of the backbone of GB1 both *in vitro* and in cell lysates by NMR (obtained by quenching the H–D exchange occurring in cell), the group was able to calculate the contribution of quinary interactions to the folding stability, and found that they are energetically comparable to those of specific protein–protein complexes (Monteith & Pielak, 2014 ▸; Monteith *et al.*, 2015 ▸; Fig. 3 ▸). Changes in the intracellular pH can modulate quinary interactions as well, as shown by observing the amide signal lineshapes of a mutant GB1 in bacteria, where the intracellular pH was controlled by changing the external buffer solution (Cohen *et al.*, 2015 ▸).

^19^F labelling has been extensively used to probe the folding thermodynamics and the conformational properties of intracellular proteins (Li *et al.*, 2010 ▸; Ye *et al.*, 2013 ▸). Li and coworkers have shown that ^19^F can be effectively utilized to investigate proteins which would not be easily detectable by ^1^H–^15^N NMR owing to severe signal broadening, such as calmodulin (CaM), both in bacteria and in *X. laevis* oocytes (Ye *et al.*, 2015 ▸). Using ^19^F labelling, Pielak and coworkers have analyzed the physiological role of protein surfaces in the folding kinetics and thermodynamics of the N-terminal domain of the signal transduction protein Drk (SH3 from *Drosophila melanogaster*), both in the bacterial cytoplasm and in buffers which mimic the intracellular environment (Smith *et al.*, 2016 ▸). Notably, the authors found that the solutes commonly used to reproduce the interior of a cell do not yield physiologically relevant information on the surface properties of proteins (whereas the properties of the hydrophobic core are well reproduced), and that electrostatic surface inter­actions are fundamental to folding stability in cells.

A typical consequence of the interactions between a soluble protein and other cellular components for the in-cell NMR spectra is the broadening of the protein signals. This effect is caused by the increased relaxation rate of NMR signals, which depends on the random reorientation (tumbling) rate of the molecule in solution. The tumbling slows down with increasing molecular size, increasing the signal broadening. Molecules that interact with other components will tumble more slowly than non-interacting molecules of the same size. Owing to the fact that interactions with the cellular environment are highly protein-dependent, proteins will experience very different signal broadening, irrespective of their size. This was clearly shown in work by the Gierasch group, in which proteins of similar size (GB1, ubiquitin, GB1–GB1 dimer and NmerA) gave rise to in-cell NMR spectra with very different signal broadening (Wang *et al.*, 2011 ▸). Unlike globular domains, unstructured proteins are less prone to the broadening of all signals, as the interacting part of the protein is rotationally independent from the rest of the polypeptide. This effect was shown by analyzing a fusion protein consisting of α-synuclein fused to GB1 through a flexible linker in *E. coli*: the NMR signals from α-synuclein were clearly visible, while those from the GB1 domain were broadened beyond detection (Barnes *et al.*, 2011 ▸). Alterations of the protein surface properties will affect the interactions with the environment, as shown in a study by Dötsch and coworkers, in which the interaction of the globular WW domain of the peptidyl-prolyl isomerase Pin1 with the components of the *X. laevis* oocytes cytosol decreased dramatically upon the phosphorylation of Pin1, which also impaired substrate recognition (Luh *et al.*, 2013 ▸).

Owing to the potential functional consequences of the interaction between a protein and the cellular environment, the question arises about which molecules in the cell are responsible for such interactions. Crowley and coworkers have investigated the case of strong interactions with cellular components by analyzing the size-exclusion chromatography (SEC) elution profile of bacterial lysates containing cytochrome *c* and a synthetic construct (ΔTat-GB1). The authors concluded that electrostatic interactions are primarily responsible for the formation of complexes in the cell lysates, which could be abolished by increasing the concentration of ions (Crowley *et al.*, 2011 ▸; Kyne *et al.*, 2015 ▸). Importantly, normal SEC elution profiles and NMR signals from ΔTat-GB1 could be recovered by pre-treating the cell lysates with RNase A, indicating that the protein interacts mainly with ribo­nucleic acids, possibly from the cellular mRNA pool.

Further support for the hypothesis that mRNA is a primary partner for the quinary interactions of proteins, both in bacteria and mammalian cells, came from Shekhtman and coworkers. By exploiting protein deuteration coupled with NMR experiments designed to detect high-molecular-weight molecules in solution, the authors showed that small proteins such as thioredoxin, FKBP, adenylate kinase and ubiquitin (ranging between 8 and 25 kDa), which are usually not detectable by in-cell NMR owing to severe line broadening, had relaxation properties compatible with complexes of about 1.2 MDa, which are consistent with the average size of the mRNAs. The same group had previously shown in yeast that ubiquitin behaves differently when the cells undergo metabolic changes induced by different growth-medium compositions (Bertrand *et al.*, 2012 ▸). These changes altered the protein local­ization from the cytosol, where the protein was free and detectable by in-cell NMR, to granular compartments, where inter­actions caused extensive line broadening. Recently, the same authors showed that different media compositions affected the total amount and average molecular weight of mRNA in yeast, and caused changes in the local­ization and interactions of ­intracellular ubiquitin and β-galactosidase (Majumder *et al.*, 2016 ▸).

**Figure 4:**
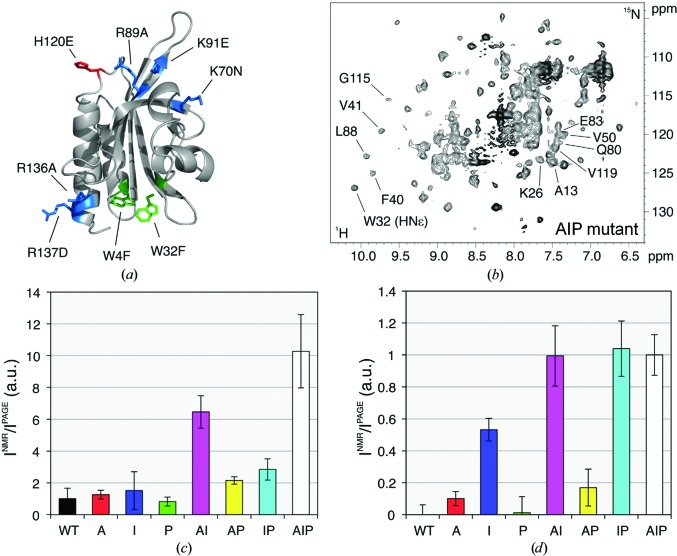
Both functional and nonspecific interactions occur in the human cytoplasm. (*a*) Mutations are introduced on the surface of human Pfn1; the mutated residues are colour-coded following the type of functional interaction that is abolished: actin (A, red), phosphoinositides (I, blue), poly-l-proline (P, green). (*b*) In-cell NMR spectrum of Pfn1 ‘full’ (AIP) mutant in human cells. (*c*, *d*) Plots of normalized NMR signal intensity for each Pfn1 mutant in human cells (*c*) and in bacteria (*d*). Adapted from Barbieri *et al.* (2015 ▸).

In addition to the quinary interactions, a protein in its physiological environment will also undergo interactions with its functional partners. While the former type of interaction is relatively weak and nonspecific, the latter is expected to be stronger and to occur only when specific proteins are present. Our research group has investigated the interactions involving the human cytoskeletal protein profilin 1 (Pfn1) in different environments, the *E. coli* and the human cytoplasm, in order to distinguish the different types of interactions (Barbieri *et al.*, 2015 ▸). By analyzing the different patterns of NMR signal recovery obtained by introducing surface mutations at different interaction sites, we showed that Pfn1 interacts with its functional partners only in the human cytoplasm (Fig. 4 ▸). Notably, further electrostatic-driven interactions occurred in both human and bacterial cells, which could be abrogated in the cell lysates by treatment with RNase A, again suggesting that mRNAs are involved in the quinary interactions.

In addition to studying the biophysical effects of the cellular environment, in-cell NMR has been successfully applied to obtain physiologically relevant information on cellular processes at the single-protein level, such as folding and maturation, post-translational modifications, misfolding and degradation.

In bacteria, Shekhtman and coworkers have developed an approach (STINT-NMR) to sequentially express two or more proteins, with only one protein being labelled (Burz *et al.*, 2006 ▸). Using this approach, they investigated processes such as the phosphorylation-dependent interaction of ubiquitin with two substrates (STAM2 and Hrs, which are components of the receptor tyrosine kinase endocytic sorting machinery), and the interaction between a prokaryotic ubiquitin-like protein (Pup) and different subunits of the proteasome of *Mycobacterium tuberculosis* (Burz & Shekhtman, 2008 ▸; Maldonado *et al.*, 2013 ▸).

In-cell NMR is also ideally applicable to protein post-translational modification events in eukaryotic cells. Among these, phosphorylation plays a major role in a wide range of cellular processes, including regulation of protein activity and signal transduction. Selenko and coworkers used time-resolved NMR to monitor a sequence of phosphorylation events catalyzed by casein kinase 2 (CK2) occurring in the regulatory region of the SV40 large T antigen both *in vitro* and in *X. laevis* oocytes and extracts (Selenko *et al.*, 2008 ▸). A sequence of stepwise phosphorylation events was observed at adjacent CK2 phosphorylation sites, which required CK2 to detach from the substrate in an intermediate step. Using a similar approach, time-resolved NMR was applied by Amata and coworkers to investigate multiple phosphorylation events on the unique domain of the nonreceptor tyrosine kinase c-Src in *X. laevis* oocytes (Amata *et al.*, 2013 ▸). By adding different inhibitors to the oocyte extracts, the authors showed that crosstalk between kinases and phosphatases took place in the extracts. Together, these studies highlight the advantage of using a time-resolved in-cell NMR approach to characterize phosphorylation events in their physiological environment in real time.

The study of cellular processes requiring correct protein folding and maturation becomes especially critical when the malfunctioning of these processes leads to pathologies, such as degenerative diseases. In this respect, in-cell NMR has proven to be a powerful technique to investigate protein-maturation events in human cells. The protein-expression approach developed in our research group is ideally suited to monitor such stepwise processes in human cells by NMR (Barbieri *et al.*, 2016 ▸). We applied this approach to the human metalloprotein copper, zinc superoxide dismutase 1 (Cu,Zn-SOD1), an evolutionarily conserved enzyme localized in the cytosol that acts as an intracellular antioxidant in all cells and tissues exposed to oxygen. In order to reach the active form, SOD1 needs to dimerize, bind one zinc and one copper ion per monomer and form an intramolecular disulfide bond. Using in-cell NMR, we observed the conformational changes of intracellular SOD1 in response to different external conditions (*i.e.* the addition of metal ions). Using *in vitro* NMR data on different metallation/redox states as a reference, we recapitulated all the maturation pathway in cultured human cells, from the apo SOD1 monomer to the zinc-bound dimer and finally the disulfide-containing, active Cu,Zn-SOD1 protein (Banci, Barbieri, Bertini *et al.*, 2013 ▸). The copper-binding and oxidation steps were achieved by co-expressing the copper chaperone for SOD1 (CCS), which is required for intracellular copper delivery to SOD1 and for disulfide-bond formation (Banci *et al.*, 2012 ▸). Unlike what is observed *in vitro*, we showed that CCS could promote disulfide formation even in the absence of copper, suggesting a previously unknown copper-independent mechanism for the oxidation of SOD1 cysteines (Banci, Barbieri, Bertini *et al.*, 2013 ▸). Understanding how human SOD1 reaches its mature form is critical, as mutations in the SOD1 gene are the root cause of a familial variant of amyotrophic lateral sclerosis (fALS), a fatal neurodegenerative disease. To date, more than 150 fALS-linked mutations have been identified, scattered throughout the amino-acid sequence of the protein. These mutant proteins cause disease onset through a toxic gain-of-function mechanism. Having monitored the maturation steps of wild-type SOD1, we sought to observe the effects of several fALS-linked mutations on the protein-maturation pathway (Luchinat *et al.*, 2014 ▸). The selected mutations do not perturb the metal-binding sites directly, but are known to destabilize the structure of apo SOD1, making the protein more prone to aggregation. Using NMR in human cells, we identified a subset of SOD1 mutations that impaired the zinc-binding step and caused the apoprotein to accumulate in a disordered, misfolded state which had not been previously characterized and could be a precursor of the toxic aggregates. Notably, co-expression of CCS rescued the maturation process of the mutant proteins, allowing them to bind zinc and eventually reach the mature, folded form (Luchinat *et al.*, 2014 ▸). This finding suggests that the metallochaperone CCS also behaves as a molecular chaperone for apo SOD1, facilitating its folding and zinc binding.

Later, Danielsson and coworkers used the SOD1 β-barrel (*i.e.* SOD1 lacking the functional metal-binding loops and the cysteine residues) to study the protein-folding thermodynamics by NMR in human cells (Danielsson *et al.*, 2015 ▸). Their data corroborate the notion that attractive interactions within the cellular environment destabilize protein folding, thereby decreasing the melting temperature. In the case of SOD1, the authors showed that in human cells a destabilizing mutation effectively causes the unfolding of the β-barrel below 37°C, which is lower than *in vitro*, thereby providing a plausible explanation for the accumulation of the disordered apo SOD1 species when fALS-linked mutations are introduced into the wild-type protein.

The redox properties of the cellular environment are also a critical factor for the correct folding and regulation of many proteins containing cysteine residues. In the cell, the redox potential is determined by the glutathione–glutathione disulfide (GSH–GSSG) redox couple; the concentrations of these two molecules determine different redox properties in the various cellular compartments. By in-cell NMR the conformations corresponding to different redox states of a protein are observed directly, and their regulation by specific cellular redox partners can be assessed. We applied in-cell NMR to investigate the redox-dependent folding and regulation of two mitochondrial proteins, Mia40 and Cox17, in human cells (Banci, Barbieri, Luchinat *et al.*, 2013 ▸; Mercatelli *et al.*, 2016 ▸). We observed that both proteins need to be kept in a reduced state by the main redox-regulating proteins of the cytoplasm (glutaredoxin 1 and thioredoxin 1) in order to be able to cross the mitochondrial outer membrane. In the presence of defective redox partners, the structural disulfide bonds of Mia40 and Cox17 are formed even in the reducing environment of the cytoplasm.

In addition to functional post-translational modifications, other chemical modifications, which can be detrimental, can occur to proteins as a consequence of oxidative stress, such as glycation and the oxidation of cysteine and methionine residues. In response to oxidative stress, cells have developed repair mechanisms to mitigate the effects of protein oxidative damage. α-Synuclein (α-Syn) is an intrinsically disordered protein implicated in the onset of Parkinson’s disease through the formation of amyloid-rich Lewy bodies. Cellular oxidative stress is causally linked to the disease, and oxidative modifications are known to promote α-Syn aggregation *in vitro*. Recently, Selenko and coworkers investigated the cellular repair mechanism of damaged α-Syn by delivering methionine-oxidized α-Syn to primary dopaminergic neurons and to other human cell lines and lysates (Binolfi *et al.*, 2016 ▸). The authors observed that while the two N-terminal methionines were reduced by the cellular methionine sulfoxide reductases in a stepwise manner, the two C-terminal methionines persisted in the oxidized form and are likely to contribute to the accumulation of permanently altered α-Syn with increased neurotoxicity.

**Figure 5:**
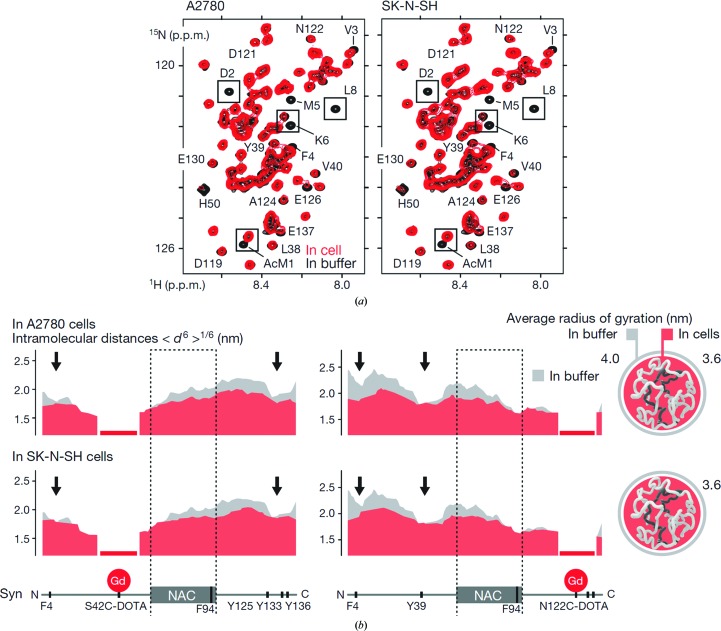
Dynamics of α-synuclein in human cell lines. (*a*) NMR spectra showing the signals from α-Syn in the cytoplasm of two human cell lines (red) and in aqueous buffer (black); decreased signal intensities correspond to regions with different protein dynamics caused by interactions with the cytosol. (*b*) Intramolecular paramagnetic relaxation enhancement profiles of α-Syn in the cytoplasm (red) and in buffer (grey); the calculated average radius of α-­Syn is smaller in the cytoplasm than in aqueous buffer. Adapted with permission from Macmillan Publishers Ltd.: Nature (Theillet *et al.*, 2016 ▸), copyright (2016).

The mechanism of α-synuclein intracellular fibril formation, like other protein misfolding and aggregation processes, has yet to be fully understood. The Selenko group has extensively characterized the intracellular dynamics of the α-Syn monomer in various human cell lines in an effort to determine how the intracellular environment affects the protein conformational space (Theillet *et al.*, 2016 ▸). Such information is critical to determine whether the cell interior modulates the initial steps of the pathogenic aggregation of α-Syn. In the cytoplasm, the protein conformation is mostly unfolded, similar to that observed *in vitro*, in contrast to previous reports of a stable helical tetramer forming inside the cells. Notably, α-Syn experiences weak hydrophobic and electrostatic quinary interactions that are lost upon cell lysis. These interactions cause α-Syn to adopt loosely compact conformations in the cell, as confirmed by NMR paramagnetic relaxation enhancement and EPR measurements (Theillet *et al.*, 2016 ▸). These conformations shield the aggregation-prone non-amyloid-β component region from exposure to the cytoplasm, presumably inhibiting spontaneous aggregation (Fig. 5 ▸).

## 6. Future perspectives   

In order to extend the applicability of in-cell NMR to increasingly challenging systems, further development is needed aimed at overcoming some longstanding practical limitations. Continuous improvements in the NMR hardware, in terms of higher field strength and advances in electronics, have increased the sensitivity of the technique. Nevertheless, the relatively short lifetime of the cells in the NMR instrument, typically a few hours, limits the type and length of the NMR experiments that can be recorded without incurring sample-stability issues. In order to ensure cell viability and stability over time, oxygen and nutrients need to be constantly replenished inside the cell sample, simultaneously removing the metabolic byproducts and stabilizing the external pH. Bioreactors designed for this purpose, which can be fitted into the NMR instruments, have been reported for in-cell NMR applications both in bacteria (Sharaf *et al.*, 2010 ▸) and in human cells (Kubo *et al.*, 2012 ▸). In both examples the cells are encapsulated within hydrogels to reduce mechanical stress, where they can still exchange nutrients and byproducts. As the general working principles of these devices are clear, a standardized design is likely to be developed in the near future that can be easily implemented in other laboratories. Similarly, improvements in sample integrity will also be needed to enable the application of solid-state NMR to intact mammalian cells.

The recent developments and applications of in-cell NMR reviewed here extensively demonstrate the unique capabilities of this approach, especially the application to human cells in order to obtain residue-level information on protein structure, dynamics, maturation, interactions and other physiological and pathological aspects. In particular, the number of applications in human cells has increased noticeably in the last few years, and we believe that the latest advances will finally allow the transition of in-cell NMR from a niche biophysical tool towards a well established cellular structural biology method.
